# Maternal Glucose during Pregnancy and after Delivery in Women with Gestational Diabetes Mellitus on Overweight Status of Their Children

**DOI:** 10.1155/2015/543038

**Published:** 2015-02-23

**Authors:** Shuang Zhang, Huikun Liu, Cuiping Zhang, Leishen Wang, Nan Li, Junhong Leng, Yi Li, Gongshu Liu, Xiangwei Fan, Zhijie Yu, Xilin Yang, Andrea A. Baccarelli, Lifang Hou, Gang Hu

**Affiliations:** ^1^Tianjin Women's and Children's Health Center, Tianjin 300070, China; ^2^Population Cancer Research Program, Dalhousie University, Halifax, NS, Canada B3H 4R2; ^3^Department of Epidemiology, School of Public Health, Tianjin Medical University, Tianjin 300070, China; ^4^Departments of Epidemiology and Environmental Health, Harvard School of Public Health, Boston, MA 02115, USA; ^5^Department of Preventive Medicine, Feinberg School of Medicine, Northwestern University, Chicago, IL 60208, USA; ^6^Chronic Disease Epidemiology Laboratory, Pennington Biomedical Research Center, 6400 Perkins Road, Baton Rouge, LA 70808, USA

## Abstract

*Objective*. To examine the association of maternal glycemia during pregnancy and after delivery with anthropometry in the offspring of mothers with gestational diabetes mellitus (GDM). *Methods*. A total of 1,263 GDM mothers and their children finished the health survey at 1–5 years after delivery. *Results*. Offspring of GDM mothers who were diagnosed with diabetes during pregnancy had higher prevalence of overweight, higher mean weight for height *Z* scores, and higher mean BMI for age *Z* scores at 1–5 years old than the offspring of GDM mothers who were diagnosed with impaired glucose tolerance (IGT) during pregnancy. Offspring of GDM mothers who developed diabetes 1–5 years after delivery had higher mean values of *Z* scores for weight for height and BMI for age at 1–5 years old than the offspring of GDM mothers who had normal glucose or prediabetes after delivery. *Conclusions*. Offspring of GDM mothers who were diagnosed with diabetes during pregnancy or after delivery had an increased risk of childhood overweight or weight gain at 1–5 years old compared with children of GDM mothers with IGT during pregnancy or with normal glucose or prediabetes after delivery.

## 1. Introduction

The prevalence of overweight/obesity has increased markedly in the last 20 years in all age groups including children younger than 5 years of age [1, 2]. Recent data have shown that excessive weight gain and/or overweight/obesity in the first several years of life are associated with increased risks of subsequent obesity and unfavorable cardiometabolic outcomes in childhood, adolescence, and adulthood [3–5]. Identifying risk factors in early prenatal and postnatal life that are related to later obesity may lead to developing early intervention strategies for primordial obesity prevention [3].

Gestational diabetes mellitus (GDM) is a risk factor which leads to certain adverse pregnancy outcome and it affects about 7% of pregnancies in the US [6]. Overall, Asian women compared with other racial/ethnic groups in the US have a much higher risk for GDM [7, 8]. In China, the prevalence of GDM has increased from 2.4% in 1999 to 8.2% in 2012 [9], now close to the US level [6]. GDM is a risk factor for obesity in offspring of GDM mothers; however, at what age these associations become apparent is unknown because very few studies have studied the GDM mothers' offspring aged younger than 5 years [10, 11]. Moreover, it is unknown how maternal glucose status during pregnancy and after delivery in GDM mothers affects overweight status of the children. The aim of the present study was to examine the relation of maternal glucose values during pregnancy and after delivery with anthropometry in the offspring of GDM mothers who participated in the Tianjin Gestational Diabetes Mellitus Prevention Program (TGDMPP) [12].

## 2. Methods

### 2.1. Tianjin GDM Screening Project

Tianjin is the fourth largest city in Northern China with over 13 million residents, and 4.3 million residents live in six central urban districts. All pregnant women who live in six urban districts have participated in the universal screening for GDM since 1999 [9, 12, 13] by using the World Health Organization (WHO) GDM criteria [14]. Following the WHO GDM screening criteria, all pregnant women at 26–30 gestational weeks participate in a 1-hour oral glucose tolerance test (OGTT) with 50 g glucose load. Women who had a glucose reading ≥7.8 mmol/L were invited to undergo a 2-hour OGTT with a 75 g glucose load at the Tianjin Women's and Children's Health Center. All women confirming either diabetes (plasma fasting glucose ≥7 mmol/L or 2-hour glucose ≥11.1 mmol/L) or impaired glucose tolerance (IGT) (2-hour glucose ≥7.8 and <11.1 mmol/L) were regarded as having GDM [9]. From December 1998 to December 2009, a total of 128,125 pregnant women took part in the GDM screening program and 6,247 were diagnosed with GDM [12]. The average proportion of screened pregnancies was over 91% during 1999–2009 [9].

### 2.2. Study Samples

**Figure 1:**
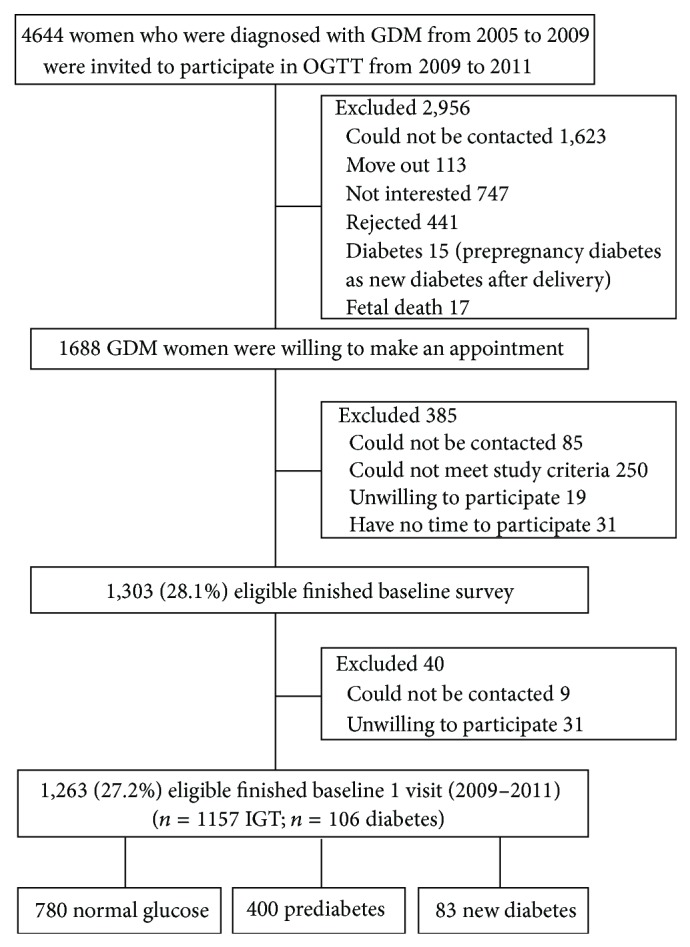
Participant flow chart.

A total of 4,644 pregnant women who were diagnosed with GDM from 2005 to 2009 and their children in six urban districts were eligible for the TGDMPP, and 1,263 GDM women and their children had completed the baseline survey for the TGDMPP from August 2009 to July 2011 (participation rate 27% of 4,644 GDM women) (Figure 1) [12, 15–17]. The sampling methods have been described previously in detail (Figure 1) [12]. Between the returned and unreturned GDM women, there were no differences at 26–30 gestational weeks of OGTT test by age (28.9 versus 28.7 years), 2-hour glucose (9.23 versus 9.16 mmol/L), fasting glucose (5.34 versus 5.34 mmol/L), and prevalence of IGT (90.9% versus 91.8%) and diabetes (9.1% versus 8.2%). The study was approved by the human subjects committee of the Tianjin Women's and Children's Health Center; informed consent was obtained for each participant.

### 2.3. Examination

At baseline survey, all GDM mothers and their children completed a self-administered questionnaire and underwent a physical examination that included anthropometric and blood pressure measurements, a 2-hour glucose 75 g OGTT (mothers only), and a fasting blood draw at the Tianjin Women's and Children's Health Center. Women were divided into three groups based on their glucose levels after delivery: normal glucose (plasma fasting glucose <5.6 mmol/L and 2-hour glucose <7.8 mmol/L), prediabetes (plasma fasting glucose 5.6–6.9 mmol/L and/or 2 h glucose 7.8–11.0 mmol/L), and diabetes (plasma fasting glucose ≥7 mmol/L or 2-hour glucose ≥11.1 mmol/L) [14]. Health workers from the Tianjin Women's and Children's Health Center collected and checked the completed questionnaire and also finished measurements. All health workers were intensively trained in meetings and in practical sessions.

At baseline survey, a questionnaire was completed by all GDM mothers. The questionnaire included questions on the mother's sociodemographics (age, marital status, education, income, and occupation); history of GDM (measured values of fasting and 2-hour glucose in the OGTT at 26–30 gestational weeks from the Tianjin Women's and Children's Health Center GDM diagnosis and treatment register system); family history of chronic diseases; medical history (hypertension, diabetes, and hypercholesterolemia); pregnancy outcomes (prepregnancy weight, weight gain in pregnancy, and number of children); dietary habits (a self-administered food frequency questionnaire (FFQ) to measure the frequency and quantity of intake of 33 major food groups and beverages during the past year) [18]; alcohol intake; smoking habits; and physical activity [19].

We also asked the GDM children's parents in advance to bring the child's birth certificate and filled in a self-administered questionnaire about the child's birth date, sex, gestational weeks of birth, birth weight, birth recumbent length, and Apgar score (above questions related to birth were copied from birth certificate), as well as the mode and duration of infant feeding (exclusive breast feeding, mixed breast and formula feeding, weaned from breast feeding, and exclusive formula feeding), health characteristics (history of illness status and current health status), dietary habits (usual habits of eating breakfast, lunch, and dinner and usual frequency of intake of vegetables, fruits, sugar-sweetened beverages, and fast food), and other lifestyle habits (duration of usual sleep and television or computer viewing). This questionnaire has been used in a longitudinal study in the same area of Tianjin [20–25].

For GDM mothers, body weight, height, waist and hip circumferences, and blood pressure were measured using the standardized protocol according to the WHO MONICA project [26]. Children's body weight was measured with a beam balance scale with participants wearing light indoor clothing without shoes. Body height was measured by a stadiometer. Weight was measured to the nearest 0.1 kg and height to the nearest 0.1 cm. Body mass index (BMI) was calculated by dividing weight in kilograms by the square of height in meters. *Z* scores for weight for age, height for age, weight for length and BMI for age, and prevalence of childhood overweight or obesity were calculated based on the standards for the WHO growth reference [27]. Children's normal weight was defined as a BMI less than the 85th percentiles for age and gender using the WHO BMI growth reference (<1.035 *Z* score), and overweight was defined as a BMI more than or equal to the 85th percentiles for age and gender specific distribution (≥1.035 *Z* score) [27]. Preterm delivery was defined as gestational weeks of delivery <37 weeks. *Z* scores for birth weight for gestational age and birth length for gestational age were calculated using our own study population means and standard deviations (*n* = 57, 454) in 2009–2011 [24]. A small-for-gestational-age infant was defined as an infant having a standardized birth weight <10th percentile, whereas a large-for-gestational-age infant was defined as an infant having a standardized birth weight >90th percentile.

### 2.4. Statistical Analyses

The general characteristics of both GDM mothers and children according to different maternal glucose concentrations (OGTT) at 26–30 gestational weeks and 1–5 years after delivery (mean 2.26 years after delivery) were compared using *t*-test and chi-square test. General linear models were used to compare the differences in *Z* scores for weight for age, height for age, weight for height, and BMI for age, changes in *Z* scores for weight for age and weight for height from birth to age of 1–5 years, and prevalence of overweight according to different maternal glucose (OGTT) concentrations at 26–30 gestational weeks and after 1–5 years of delivery. Logistic regression was used to compare the relative risk of overweight according to different maternal glucose concentrations at 26–30 gestational weeks and 1–5 years after delivery. All analyses were adjusted for maternal age, prepregnancy BMI, weight gain during pregnancy, family history of diabetes, marital status, education, income, gestational age at delivery, and infant feeding. To explore the potential mediating effort, in multivariable model 2, we additionally adjusted for birth weight for gestational age *Z* score or birth weight for length for gestational age *Z* score in the analysis of change in weight for age *Z* score and weight for height *Z* score. All statistical analyses were performed with PASW for Windows, version 20.0 (Statistics 20, SPSS, IBM, USA).

## 3. Results

Of 1,263 women who were diagnosed with GDM from 2005 to 2009, 1157 were diagnosed with IGT and 106 with diabetes based on their OGTT glucose concentrations at 26–30 gestational weeks. After a mean 2.26-year (27.2 months) delivery of 1263 GDM mothers, 83 were diagnosed with type 2 diabetes (6.6%) and 400 with prediabetes (31.7%). GDM women with diagnosed diabetes during pregnancy or after delivery were slightly older, their prepregnancy and current BMI and fasting and 2-hour glucose in the OGTT at 26–30 gestational weeks were higher, and they had less gestational weight gain as compared with GDM women with IGT during pregnancy or with normal glucose after delivery (Table 1). The offspring of GDM mothers diagnosed with diabetes during pregnancy or after delivery had shorter gestational age and were more often premature and large for gestational age at delivery compared with those of GDM mothers with IGT during pregnancy or with normal glucose after delivery. However, offspring of GDM mothers diagnosed with diabetes after delivery had higher mean values of *Z* scores of birth weight for gestational age and birth weight for length for gestational age than those of GDM mothers with normal glucose after delivery.

Offspring of GDM mothers who were diagnosed with diabetes at 26–30 gestational weeks had higher mean values of weight for height *Z* scores, BMI for age *Z* scores, changes in *Z* score for weight for height from birth to age of 1–5 years, and prevalence of childhood overweight [27] than the offspring of GDM mothers who were diagnosed with IGT at 26–30 gestational weeks (Table 2). Offspring of GDM mothers who were diagnosed with diabetes at a mean of 2.26 years after delivery had higher mean values of *Z* scores for weight for height and BMI for age at 1–5 years than the offspring of GDM mothers who were with normal glucose or prediabetes after delivery (Table 2).

We additionally assessed joint status of maternal OGTT glucose at 26–30 gestational weeks (IGT and diabetes) and diabetes status 1–5 years after delivery (nondiabetes and diabetes) with anthropometry in offspring of GDM mothers (Table 3). Offspring of GDM mothers who were diagnosed with diabetes at 26–30 gestational weeks and with nondiabetes at a mean of 2.26 years after delivery, offspring of GDM mothers who were diagnosed with IGT at 26–30 gestational weeks and with diabetes at a mean of 2.26 years after delivery, and offspring of GDM mothers who were diagnosed with diabetes both at 26–30 gestational weeks and at a mean of 2.26 years after delivery all had higher mean values of weight for height *Z* scores, BMI for age *Z* scores, and change in weight for height *Z* score from birth to 1–5 years than the offspring of GDM mothers who were diagnosed with IGT at 26–30 gestational weeks and with nondiabetes at a mean of 2.26 years after delivery.

## 4. Discussion

The present study indicated that the offspring of GDM mothers who were diagnosed with diabetes at 26–30 gestational weeks or 1–5 years after delivery had higher mean values of *Z* scores for weight for height and BMI for age at 1–5 years old than the offspring of GDM mothers who were diagnosed with IGT during pregnancy or who were with normal glucose or prediabetes after delivery. Offspring of GDM mothers who were diagnosed with diabetes at 26–30 gestational weeks had an increased risk of overweight at 1–5 years old compared with those children of GDM mothers with IGT during pregnancy.

GDM is increasingly common around the world. The exposure to diabetes during pregnancy has been found as an important prenatal predictor of obesity from childhood to younger adults [28]. However, at what age these associations become apparent is unknown because very few studies are targeted at the GDM's offspring aged younger than 5 years [10, 11]. In the study of the Diabetes in Pregnancy Center at Northwestern University in Chicago, diabetes during pregnancy, including both GDM and insulin-treated preexistent diabetes, was associated with increased BMI of the offspring at birth and after the age of 5 years [29, 30]. The offspring of Pima Indian women with preexistent diabetes and GDM were heavier at birth and had much higher rates of obesity at age of 5–19 years than the offspring of prediabetic or nondiabetic women [31, 32]. However, other studies did not find a clear association between maternal GDM and obesity in the offspring of more than 5 years old [33–35]. Two recent studies found no association between maternal glucose during pregnancy and obesity in the 2-year-old offspring [10, 36]. However, in a multiethnic population from Colorado, exposure to diabetes in utero was associated with an altered growth trajectory in children from 2 years of age through 13 years [10].

No previous studies have evaluated maternal glucose status during pregnancy and after delivery in GDM mothers on overweight/obesity status of their children aged less than 5 years. The present study, for the first time, found that offspring of GDM mothers diagnosed with diabetes at 26–30 gestational weeks had the same mean values of birth weight and birth length, higher mean values of *Z* scores of weight for height and BMI for age, higher mean values of changes in *Z* scores for weight for height from birth to age of 1–5 years, and higher prevalence of childhood overweight than the offspring of GDM mothers who were diagnosed with IGT during pregnancy. Offspring of GDM mothers who were diagnosed with diabetes after delivery had higher mean values of *Z* scores for birth weight for gestational age, birth length for gestational age, weight for height, and BMI for age at 1–5 years old than the offspring of GDM mothers who were with normal glucose or prediabetes after delivery. These findings suggest that maternal high glucose status during pregnancy in GDM mothers and diabetes diagnoses in GDM mothers after delivery are very important risk factors for children's later overweight risk. The major reasons of diabetes diagnoses in GDM mothers after delivery as a risk factor for their children being overweight are that these GDM mothers already had higher levels of prepregnancy BMI and fasting and 2-hour glucose in the OGTT at 26–30 gestational weeks compared with GDM women who did not develop diabetes after delivery (Table 1).

One strength of this study is standardized screening of GDM during pregnancy and standardized testing of diabetes status among GDM mothers after delivery. Other strengths of this study include a large number of GDM mother-child pairs, its prospective study design, and adjustment for multiple prenatal and perinatal factors in analyzing the association of maternal glucose during pregnancy and after delivery in GDM women with overweight status of their children. A limitation of our study is that we only include GDM mothers and their children and do not include a normal glucose control group during pregnancy, which may reduce generalizability. Another limitation of this study is that we only used WHO's criteria to screen and diagnose GDM from 2005 to 2009. In 2010, the International Association of Diabetes and Pregnancy Study Groups (IADPSG) recommended new criteria to define GDM with a much lower cutoff point for the fasting glucose than previous criteria for diagnosis of GDM and an additional measurement of 1-hour glucose [37]. Since we did not measure 1-hour glucose during an OGTT of GDM screening, we might miss some GDM cases in the present analysis.

In summary, we found that offspring of GDM mothers diagnosed with diabetes during pregnancy or 1–5 years after delivery are associated with an increased risk of childhood overweight or weight gain compared with those of GDM mothers with IGT during pregnancy or with normal glucose after delivery. These findings suggest that maternal glucose status during pregnancy in GDM women and diabetes diagnoses in GDM mothers after delivery might lead to their children being overweight. More studies are needed to confirm our finding because we did not include a non-GDM control group.

## Figures and Tables

**Table 1:** Characteristics of 1263 gestational diabetes mellitus (GDM) mother-child pairs in Tianjin Gestational Diabetes Mellitus Prevention Program.

	2-hour glucose level at 26–30 gestational weeks of OGTT			*P* for difference	Maternal glycemic status after 1–5 years of delivery^a^			*P* for difference
	7.8–8.69 (IGT)	8.7–11.09 (IGT)	≥11.1 (DM)		Normal glucose	Prediabetes^a^	Diabetes^a^	
Number of subjects	576	581	106		780	400	83	
Maternal characteristics								
Age (years)	32.0 (3.3)	32.6 (3.5)	33.4 (4.0)	<0.001	32.3 (3.5)	32.5 (3.5)	32.7 (3.8)	0.59
Prepregnancy BMI (kg/m^2^)	22.8 (3.2)	23.2 (3.4)	24.2 (3.5)	<0.001	22.5 (3.0)	23.9 (3.4)	25.6 (3.7)	<0.001
Fasting glucose at 26–30 gestational weeks (mmol/L)	5.1 (0.6)	5.3 (0.7)	6.4 (1.3)	<0.001	5.2 (0.7)	5.5 (0.8)	6.0 (1.2)	<0.001
2-hour glucose at 26–30 gestational weeks (mmol/L)	8.2 (0.3)	9.6 (0.6)	12.3 (1.1)	<0.001	8.9 (1.1)	9.3 (1.3)	10.5 (1.7)	<0.001
Gestational weight gain (kg)	17.2 (6.1)	16.7 (6.1)	15.7 (5.4)	0.053	17.2 (6.0)	16.5 (6.0)	14.6 (5.9)	<0.001
Current BMI (kg/m^2^)	24.1 (3.8)	24.1 (4.0)	24.9 (3.9)	0.14	23.2 (3.4)	25.4 (4.1)	27.3 (4.0)	<0.001
Current smoking (%)	3.3	1.5	0.9	0.12	2.3	2.2	2.4	0.19
Prepregnancy overweight (%)^b^	32.6	35.3	45.3	0.042	28.3	41.8	63.9	<0.001
Education (%)				0.074				0.046
<13 years	23.4	19.3	31.1		19.7	24.8	32.5	
13–16 years	69.3	72.6	63.2		72.2	68.5	61.4	
≥16 years	7.3	8.1	5.7		8.1	6.8	6.0	
Family income (yuan/month)				0.13				0.014
<5000	25.9	28.6	31.1		25.3	29.2	41.0	
5000–8000	39.2	36.3	26.4		36.9	36.8	36.1	
≥8000	34.9	35.1	42.5		37.8	34.0	22.9	
Married (%)	99.0	99.1	98.1	0.63	99.0	99.0	98.9	0.99
Family history of diabetes (%)	32.3	36.1	48.1	0.007	31.4	38.5	57.8	<0.001
Children characteristics								
Sex (boys %)								
Age at baseline survey (months)	28.0 (10.6)	26.8 (10.3)	28.0 (10.6)	0.10	27.1 (10.4)	27.3 (10.2)	31.6 (11.4)	0.001
Age range (months)	13.6–59.0	14.1–59.0	15.4–59.0		13.6–59.0	14.6–59.0	14.7–58.6	
Gestational age at delivery (weeks)	39.1 (1.5)	39.0 (1.5)	38.6 (1.8)	0.008	39.1 (1.5)	39.1 (1.4)	38.5 (1.8)	0.008
Birth weight (g)	3541 (501)	3550 (541)	3540 (559)	0.95	3512 (491)	3598 (557)	3601 (635)	0.018
Birth recumbent length (cm)	50.8 (1.9)	50.7 (2.1)	51.0 (2.3)	0.28	50.6 (2.0)	50.9 (2.0)	51.1 (2.5)	0.010
Birth weight for gestational age *Z* score	0.38 (1.2)	0.44 (1.2)	0.57 (1.2)	0.30	0.34 (1.1)	0.52 (1.3)	0.76 (1.3)	0.001
Birth weight for length for gestational age *Z* score	0.33 (1.2)	0.41 (1.2)	0.46 (1.2)	0.40	0.31 (1.1)	0.46 (1.3)	0.63 (1.2)	0.014
Preterm (%)	3.6	4.8	9.4	0.034	4.1	4.2	12.0	0.004
Small for gestational age (%)	5.7	4.5	6.6	0.51	4.6	6.5	4.8	0.38
Large for gestational age (%)	18.9	21.3	24.5	0.34	16.5	25.8	32.5	<0.001
Breast feeding (%)				0.046				0.048
Exclusive breast feeding	43.9	38.6	35.8		41.9	40.0	33.7	
Mixed feeding	41.5	43.2	40.6		42.6	42.0	39.8	
Weaned from breast feeding	1.2	3.3	1.9		2.2	2.8	0	
Exclusive formula feeding	13.4	15.0	21.7		13.3	15.2	26.5	

Baseline characteristics represent mean (SD) or percentage.

^a^Prediabetes was defined as plasma fasting glucose 5.6–6.9 mmol/L and/or 2 h glucose 7.8–11.0 mmol/L; diabetes was defined as fasting glucose ≥7.0 mmol/L or 2 h glucose ≥11.1 mmol/L.

^b^Overweight was defined as body mass index more than 24 kg/m^2^.

**Table 2:** Mean values of *Z* scores for weight, length/height, body mass index, prevalence, and relative risk of overweight among offspring of GDM mothers at age of 1–5 years according to strata of maternal OGTT glucose at 26–30 gestational weeks and maternal diabetes status after 1–5 years of delivery.

	2-hour glucose level at 26–30 gestational weeks of OGTT			*P* for difference	Maternal glycemic status after 1–5 years of delivery^a^			*P* for difference
	7.8–8.69 (IGT)	8.7–11.09 (IGT)	≥11.1 (DM)		Normal glucose	Prediabetes^a^	Diabetes^a^	
Number of subjects	576	581	106		780	400	83	
Weight for age *Z* score								
Multivariable model 1^b^	0.56 (0.04)	0.59 (0.04)	0.79 (0.09)	0.083	0.57 (0.04)	0.60 (0.05)	0.79 (0.11)	0.15
Multivariable model 2^c^	0.57 (0.04)	0.58 (0.04)	0.77 (0.09)	0.12	0.58 (0.03)	0.58 (0.05)	0.73 (0.11)	0.40
Length/height for age *Z* score								
Multivariable model 1^b^	0.47 (0.04)	0.58 (0.04)	0.58 (0.10)	0.18	0.51 (0.04)	0.55 (0.05)	0.56 (0.12)	0.82
Multivariable model 2^c^	0.48 (0.04)	0.57 (0.04)	0.57 (0.10)	0.27	0.52 (0.04)	0.54 (0.05)	0.51 (0.11)	0.96
Weight for length/height *Z* score								
Multivariable model 1^b^	0.42 (0.04)	0.39 (0.04)	0.68 (0.10)	0.029	0.40 (0.04)	0.42 (0.05)	0.69 (0.12)	0.045
Multivariable model 2^c^	0.42 (0.04)	0.39 (0.04)	0.67 (0.10)	0.028	0.41 (0.04)	0.41 (0.05)	0.67 (0.11)	0.096
Body mass index for age *Z* score								
Multivariable model 1^b^	0.39 (0.04)	0.34 (0.04)	0.63 (0.10)	0.028	0.37 (0.04)	0.37 (0.05)	0.65 (0.12)	0.060
Multivariable model 2^c^	0.40 (0.04)	0.34 (0.04)	0.63 (0.10)	0.026	0.37 (0.04)	0.37 (0.05)	0.63 (0.12)	0.10
Change in weight for age *Z* score								
Multivariable model 1^b^	0.17 (0.05)	0.15 (0.05)	0.27 (0.12)	0.64	0.22 (0.05)	0.10 (0.06)	0.08 (0.15)	0.28
Multivariable model 2^c^	0.15 (0.04)	0.16 (0.04)	0.35 (0.09)	0.12	0.16 (0.03)	0.16 (0.05)	0.31 (0.11)	0.40
Change in weight for length/height *Z* score								
Multivariable model 1^b^	0.08 (0.06)	0.02 (0.06)	0.25 (0.14)	0.15	0.08 (0.05)	−0.02 (0.07)	0.09 (0.16)	0.53
Multivariable model 2^c^	0.04 (0.04)	0.01 (0.04)	0.29 (0.10)	0.039	0.03 (0.04)	0.04 (0.05)	0.28 (0.11)	0.10
Prevalence of overweight^d^ (%)								
Multivariable model 1^b^	23.1	20.7	31.2	0.055	22.3	22.5	26.5	0.70
Multivariable model 2^c^	23.2	20.6	31.0	0.053	22.5	22.3	25.8	0.78
Relative risk of overweight								
Multivariable model 1^b^	1.00	0.87 (0.65–1.16)	1.56 (0.99–2.50)	0.047	1.00	1.01 (0.75–1.36)	1.25 (0.73–2.14)	0.72
Multivariable model 2^c^	1.00	0.86 (0.64–1.15)	1.55 (0.98–2.49)	0.046	1.00	0.99 (0.73–1.33)	1.20 (0.70–2.06)	0.78

Data represent mean (SE) or percentage.

^a^Prediabetes was defined as plasma fasting glucose 5.6–6.9 mmol/L and/or 2 h glucose 7.8–11.0 mmol/L; diabetes was defined as fasting glucose ≥7.0 mmol/L or 2 h glucose ≥11.1 mmol/L.

^b^Multivariable model 1 was adjusted for maternal age, prepregnancy BMI, weight gain during pregnancy, family history of diabetes, marital status, education, income, gestational age at delivery, and infant feeding.

^c^Multivariable model 2 was adjusted for above variables and also birth weight for gestational age *Z* score or birth weight for length for gestational age *Z* score in the analysis of change in weight for age *Z* score and weight for length/height *Z* score.

^d^Overweight is defined as a body mass index more than the 85th percentiles for age and gender using the WHO growth reference.

**Table 3:** Mean values of *Z* scores for weight, length/height, body mass index, prevalence, and relative risk of overweight among offspring of GDM mothers at age of 1–5 years according to joint status of maternal OGTT glucose at 26–30 gestational weeks and diabetes status after 1–5 years of delivery.

	Maternal 2-hour glucose level at 26–30 gestational weeks of OGTT and glycemic status after 1–5 years of delivery^a^				*P* for difference
	IGT during pregnancy/non-DM after delivery	DM during pregnancy/non-DM after delivery	IGT during pregnancy/DM after delivery	DM during pregnancy/DM after delivery	
Number of subjects	1102	78	55	28	
Weight for age *Z* score					
Multivariable model 1^b^	0.57 (0.03)	0.77 (0.11)	0.74 (0.13)	0.81 (0.19)	0.12
Multivariable model 2^c^	0.57 (0.03)	0.75 (0.11)	0.69 (0.13)	0.78 (0.18)	0.24
Length/height for age *Z* score					
Multivariable model 1^b^	0.52 (0.03)	0.53 (0.12)	0.52 (0.14)	0.63 (0.19)	0.96
Multivariable model 2^c^	0.53 (0.03)	0.52 (0.11)	0.47 (0.14)	0.63 (0.19)	0.93
Weight for length/weight *Z* score					
Multivariable model 1^b^	0.39 (0.03)	0.68 (0.11)	0.66 (0.14)	0.66 (0.19)	0.019
Multivariable model 2^c^	0.39 (0.04)	0.67 (0.12)	0.62 (0.14)	0.66 (0.19)	0.029
Body mass index for age *Z* score					
Multivariable model 1^b^	0.35 (0.03)	0.65 (0.12)	0.61 (0.14)	0.61 (0.20)	0.023
Multivariable model 2^c^	0.36 (0.04)	0.64 (0.12)	0.59 (0.14)	0.61 (0.20)	0.034
Change in weight for age *Z* score					
Multivariable model 1^b^	0.17 (0.04)	0.24 (0.15)	0.06 (0.18)	0.25 (0.25)	0.85
Multivariable model 2^c^	0.15 (0.03)	0.33 (0.11)	0.26 (0.13)	0.36 (0.18)	0.24
Change in weight for length/height *Z* score					
Multivariable model 1^b^	0.03 (0.04)	0.22 (0.16)	0.04 (0.19)	0.27 (0.27)	0.58
Multivariable model 2^c^	0.02 (0.03)	0.28 (0.12)	0.24 (0.14)	0.28 (0.19)	0.040
Prevalence of overweight^d^ (%)					
Multivariable model 1^b^	21.7	33.7	25.8	23.8	0.10
Multivariable model 2^c^	21.7	33.4	25.0	23.8	0.12
Relative risk of overweight					
Multivariable model 1^b^	1.00	1.89 (1.14–3.13)	1.23 (0.66–2.32)	1.16 (0.49–2.76)	0.095
Multivariable model 2^c^	1.00	1.87 (1.13–3.11)	1.17 (0.62–2.21)	1.16 (0.49–2.76)	0.11

^a^IGT, impaired glucose tolerance, was defined as 2-hour glucose >7.7 but <11.1 mmol/L; diabetes was defined as fasting glucose ≥7.0 mmol/L or 2 h glucose ≥11.1 mmol/L.

^b^Multivariable model 1 was adjusted for maternal age, prepregnancy BMI, weight gain during pregnancy, family history of diabetes, marital status, education, income, gestational age at delivery, and infant feeding.

^c^Multivariable model 2 was adjusted for above variables and also birth weight for gestational age *Z* score or birth weight for length for gestational age *Z* score in the analysis of change in weight for age *Z* score and weight for length/height *Z* score.

^d^Overweight is defined as a body mass index more than the 85th percentiles for age and gender using the WHO growth reference.
